# miR-302a inhibits human HepG2 and SMMC-7721 cells proliferation and promotes apoptosis by targeting *MAP3K2* and *PBX3*

**DOI:** 10.1038/s41598-018-38435-0

**Published:** 2019-02-14

**Authors:** Meng Wang, Guoyue Lv, Chao Jiang, Shuli Xie, Guangyi Wang

**Affiliations:** Department of Hepatobiliary and Pancreatic Surgery, The First Hospital of Jilin University, 71 Xinmin Street, Changchun, 130021 Jilin, China

## Abstract

Hepatocellular carcinoma (HCC) is the most common liver cancer and has a poor prognosis. miR-302a is an important regulator of tumor occurrence and deterioration, while *MAP3K2* and *PBX3* genes are involved in cancer cell proliferation and apoptosis. In this study, the expression of miR-302a and *MAP3K2*/*PBX3* were evaluated by qPCR in liver cancer cell lines. Next, the target relationship between miR-302a and *MAP3K2*/*PBX3* was verified using luciferase assays. Meanwhile, the expression correlation between miR-302a and target genes was analyzed in cancer tissue and para-cancerous tissue. In addition, an increased miR-302a level in HepG2 cells and SMMC-7721 cells were achieved through transfection with miR-302a mimics, and the effects on HepG2 cell and SMMC-7721 cell proliferation, apoptosis and MAPK pathways were determined using MTT, flow cytometry, qPCR and western blot assays. The results showed that liver cancer cell lines exhibited low miR-302a expression and *MAP3K2* and *PBX3* were confirmed to be the target genes of miR-302a. Meanwhile, the HE results showed that cells became enlarged with loose cytoplasm and formed balloon-like lesions in HCC specimens and we found a significant negative correlation between miR-302a and *MAP3K2*/*PBX3* expression. In addition, treatment with miR-302a mimics inhibited HepG2 cells and SMMC-7721 cells proliferation and increased the apoptosis rate. Further research revealed that the *MAPK* key factors p-p38, p-ERK1/2 and p-JNK were significantly reduced in miR-302a transfected cells and *MAP3K2*/*PBX3* silenced cells. Besides, *MAP3K2* and *PBX3* overexpression in miR-302a mimics-treated cells exerted the opposite effects. In conclusion, miR-302a inhibited proliferation and promoted apoptosis in human hepatoma cells by targeting *MAP3K2* and *PBX3*.

## Introduction

HCC is one of the most frequently diagnosed cancers and a major cause of cancer-related death worldwide. Local recurrence and distant metastasis result in poor prognosis^1^. Various etiologies are associated with the development of HCC, including Hepatitis B and C virus infections, immune system imbalance, gene mutation and harsh living conditions^2–5^. To date, surgical resection and orthotopic liver transplantation are considered the only possibly curative therapies for early-stage HCC. Therefore, biomarkers and evaluation of their clinical utility in surveillance and early diagnosis of HCC are urgently needed^6^.

miRNAs, which are small, non-encoding single-stranded RNAs approximately 22 nucleotides in length, are crucial molecules that act as tumor suppressors or oncogenes in human cancer progression. miR-302a, a member of the miR-302 family, is located on chromosome 4q25, has a mature sequence approximately 23 bp in length and participates in the occurrence, development and deterioration of various cancer types. Studies have shown that miR-302 family members are tumor repressors in human cancer and suppress proliferation and induce apoptosis in human melanoma Colo-829 cells, prostate cancer PC3 cells, human breast cancer MCF7 cells, hepatocellular carcinoma HepG2 cells, and embryonal teratocarcinoma Tera-2 cells^7–9^. In addition, in brain cancer, miR-302 coordinately acts with the MTKI sunitinib to decrease GBM cell viability^10^. Furthermore, the miR-302a expression level is significantly lower in gastric cancer (GC), and its low expression is frequently accompanied by positive lymph node metastasis, advanced TNM stage and great invasion depth and significantly associated with shorter disease-free and overall survival of GC patients^11^. In addition, miR-302a can act as a tumor suppressor to inhibit glioma cell and colon cancer cell proliferation^12–14^. Recently, a new miR-302a/VEGFA axis is identified and found to be involved in HCC formation and progression. miR-302a is expressed at lower levels in HCC tissues and cell lines than matched NATs. Reduced miR-302a expression is correlated with tumor-node-metastasis stage and lymph node metastasis in patients with HCC^15^.

Bioinformatic prediction and analysis results showed that mitogen-activated protein kinase kinase2 (*MAP3K2, MEKK2*) and pre-B-cell leukemia homeobox 3 (*PBX3*) were possible target genes of miR-302a. Studies have confirmed that *MAP3K2* is involved in several cancer types and is closely relate to the risk of mortality. In breast tumor cells and lung cancer cells, *MAP3K2* plays a pivotal role in promoting cell proliferation^16,17^. Meanwhile, *MAPK* signaling genes can increase the risk of colorectal cancer and have been associated with poor prognosis in squamous cell carcinoma^18,19^. *PBX3* is also found to participate in the regulation of a variety of tumors, such as glioma^15^, gastric cancer (GC)^20^ and invasive prostate cancer^21^, and elevated *PBX3* expression significantly promote tumor cell proliferation. Furthermore, both *MAP3K2* and *PBX3* participate in HCC regulation^20,22–24^. *MAP3K2* may be involved in the regulation of *MAPK* signaling pathway in cancer deterioration by KEGG analysis. And it is well known that *MAPK* pathways regulate cellular functions including cell proliferation, differentiation, migration, and apoptosis^25,26^. MEKK2 is a serine/threonine kinase that functions as a MAPK kinase kinase (MAP3K) to regulate activation of MAPKs^7,27^. Meanwhile, the MAPK kinase kinase MEKK2 is essential for activation of c-Jun N-terminal kinase (JNK) and extracellular signal-regulated kinase (ERK)^28^. Furthermore, MEKK2 immunoprecipitates activated c-Jun in an IL-1 dependent manner and this activity is inhibited by the selective JNK inhibitor SP600125. Of interest, MEKK1 immunoprecipitates from IL-1-stimulated FLS appeared to activate c-Jun through the JNK pathway and TAK1 activation of c-Jun is dependent on JNK, ERK, and p38^29^. In addition, *PBX3* knockdown inhibits *MAPK* pathway activation in glioma cells. As shown in research, *PBX3* knockdown significantly reduces the phosphorylation level of p38 and ERK1/2. Taken together, the results indicate miR-320 may suppress glioma cell growth through targeting *PBX3* and regulating *MAPK* pathway^30^. However, the role of miR-302a in HCC pathogenesis and progression through the target genes and its impact on growth-regulatory pathways remains unclear.

In this study, the target relationship between miR-302a and *MAP3K2/PBX3* was predicted and verified. And miR-302a, *MAP3K2* and *PBX3* expression levels were detected in liver cancer cells and tissues. In addition, the effect of miR-302a on *MAPK* signaling pathways, cell proliferation and apoptosis was examined in HepG2 cells and SMMC-7721 cells. The data will lay a theoretical foundation for HCC early diagnosis and treatment.

## Results

### *MAP3K2* and *PBX3* are target genes of miR-302a

**Figure 1:**
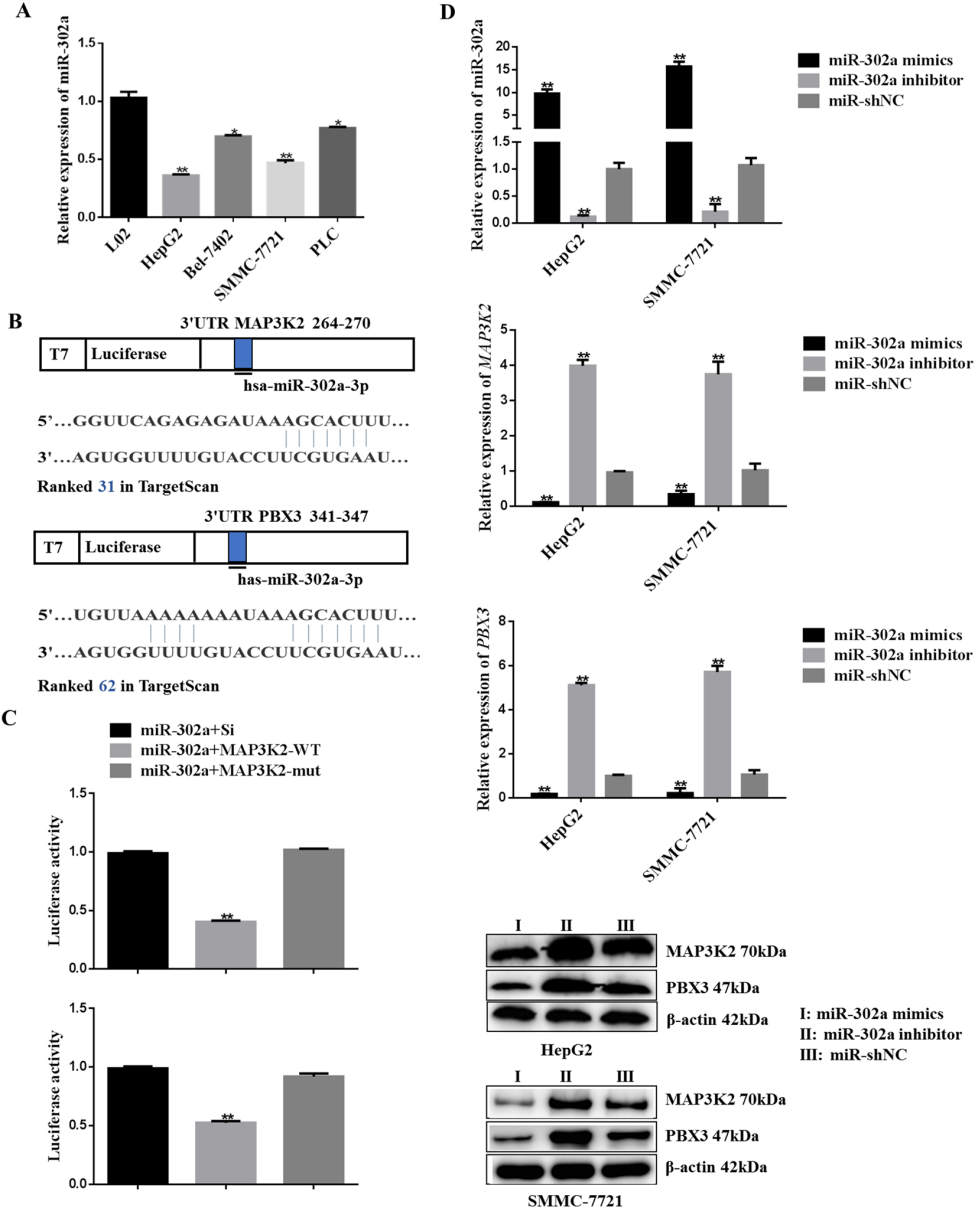
*MAP3K2* and *PBX3* are targets of miR-302a. (**A**) The expression of miR-302a were detected in HCC cells (HepG2, Bel-7402, SMMC-7721 and PLC) and a human immortalized normal liver epithelial cells (L02). (**B**) The seed-recognition sites were predicted in the *MAP3K2* and *PBX3* 3′UTRs. (**C**) Dual-luciferase reporter assays were performed in HepG2 cells co-transfected with miR-302a mimics and *MAP3K2-3′UTR* or *PBX3-3′UTR* (**P < 0.01). (**D**) The effect of miR-302a were detected on target genes MAP3K2 or PBX3 in HepG2 and SMMC-7721 cells that transfected with miR-302a mimics, inhibitor and miR-shNC. Statistical analysis was conducted using a Fisher’s least significant difference (LSD) method of two-way analysis of variance (ANOVA).

First, we examined the expression of miR-302a in normal liver cells L02 and liver cancer cells. Results showed that low miR-302a expression was found in liver cancer cell lines (HepG2, Bel-7402, SMMC-7721 and PLC) compared with control group (L02) cells (Fig. 1A) (P < 0.01). The result suggesting that miR-302a might be involved in HCC.

After that, according to bioinformatics software prediction, 1012 possible target genes were searched. First, *MAP3K2* and *PBX3* were predicted to strongly bind with miR-302a. In addition, GO analysis and KEGG analysis showed that *MAP3K2* and *PBX3* participated in cancer regulation. Therefore, *MAP3K2* and *PBX3* were selected from the pool of 1012 possible targets. We identified miR-302a binding sites within the *3′UTRs* of *MAP3K2* and *PBX3*, as shown in Fig. 1B. For validation of the potential targets, luciferase activity was analyzed. As shown in Fig. 1C, compared with the *miR-302a mimics* + *Report-si* group, luciferase activity was lower in cells co-transfected with *miR-302a mimics* + *MAP3K2-WT* vectors (P < 0.01), and no significant difference was observed in the *miR-302a mimics* + *MAP3K2-mut* group. Analogously, luciferase activity was significantly lower in the *miR-302a mimics* + *PBX3-WT* transfected cells than in the *miR-302a mimics* + *Report-si/PBX3-mut* group (P < 0.01). As indicated above, *MAP3K2* and *PBX3* were both target genes of miR-302a. Moreover, miR-302a significantly inhibited *MAP3K2* and *PBX3* mRNA and protein expression levels (Fig. 1D, P < 0.01) (Supplementary Figure S4).

### Morphology observation and correlation between miR-302a and target genes in HCC tissues

**Figure 2:**
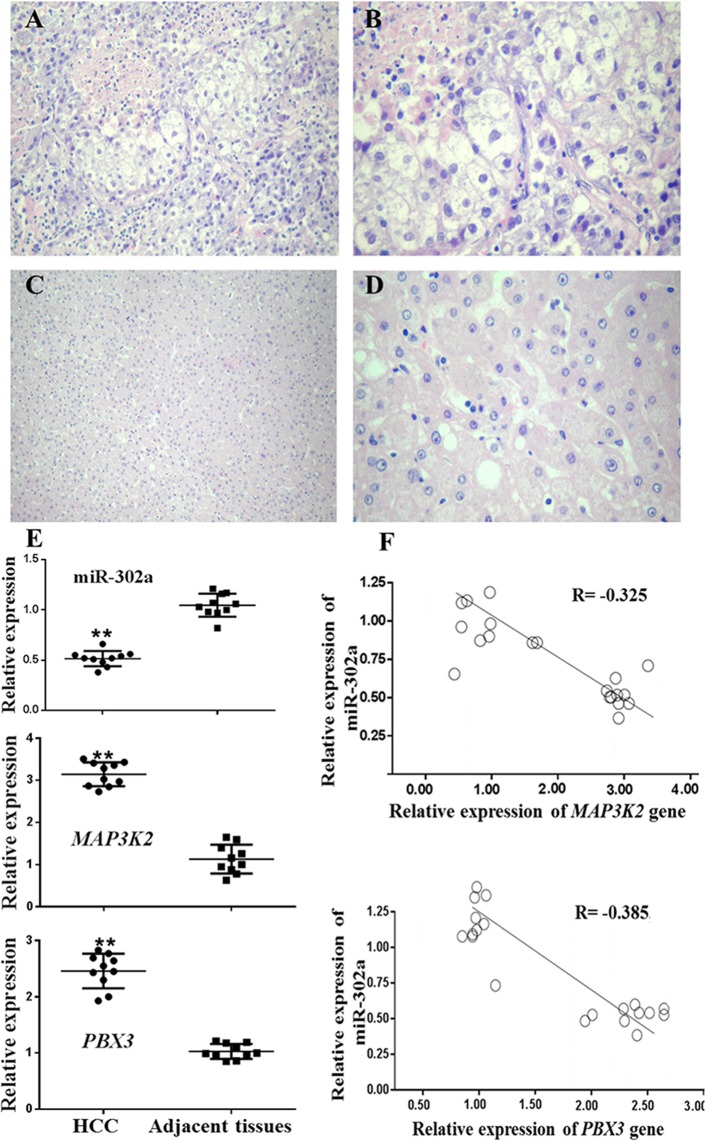
Morphology observation and correlation between miR-302a and target genes analyzed in HCC tissues. (**A**–**D**) The HE-stained HCC tissues in A (×200) and B (×400) showed a typical HCC morphology, and the adjacent tissues showed a normal liver cell morphology (C ×200 and D ×400). (**E**) The expression of miR-302a, *MAP3K2* and *PBX3* were detected in 10 HCC tissues and 10 adjacent tissues by qPCR (**P < 0.01). (**F**) Linear correlation was applied to analyze the correlation between the miR-302a and *MAP3K2* or *PBX3* mRNA expression levels (**P < 0.01). Statistical analysis was conducted using Student’s t-test and Fisher’s least significant difference (LSD) method of two-way analysis of variance (ANOVA).

The morphology of HCC and adjacent tissues was analyzed under a microscope, and representative images were shown in Fig. 2. In Fig. 2A and B, the HE-stained HCC tissues showed a typical HCC morphology. The cells in HCC specimens were enlarged with loose cytoplasm and formed balloon-like lesions. The adjacent tissues showed normal liver cell morphology and mild hepatic steatosis with a small amount of inflammatory cell infiltration (Fig. 2C and D).

Then, we examined the expression of mature miR-302a and *MAP3K2*/*PBX3* in HCC and adjacent tissues. As shown in Fig. 2E, in 10 HCC patients, the expression of mature miR-302a was significantly lower in HCC specimens than in adjacent tissues from the same patients (P < 0.01). The analysis of miR-302a expression in clinical cases was convincing due to primer verification and the relative expression detection results of miR-302b, miR-302c and miR-302d (Supplementary Figures S1 and S2). However, the target gene mRNA expression showed the opposite trend (Fig. 2E) (P < 0.01). The data showed that miR-302a was negatively correlated with *MAP3K2* mRNA in HCC tissues (Fig. 2F) (R = −0.325, P < 0.05) and was also negatively correlated with *PBX3* (Fig. 2F) (R = −0.385, P < 0.05).

### miR-302a promotes HepG2 cells and SMMC-7721 cells apoptosis and inhibits cell proliferation

**Figure 3:**
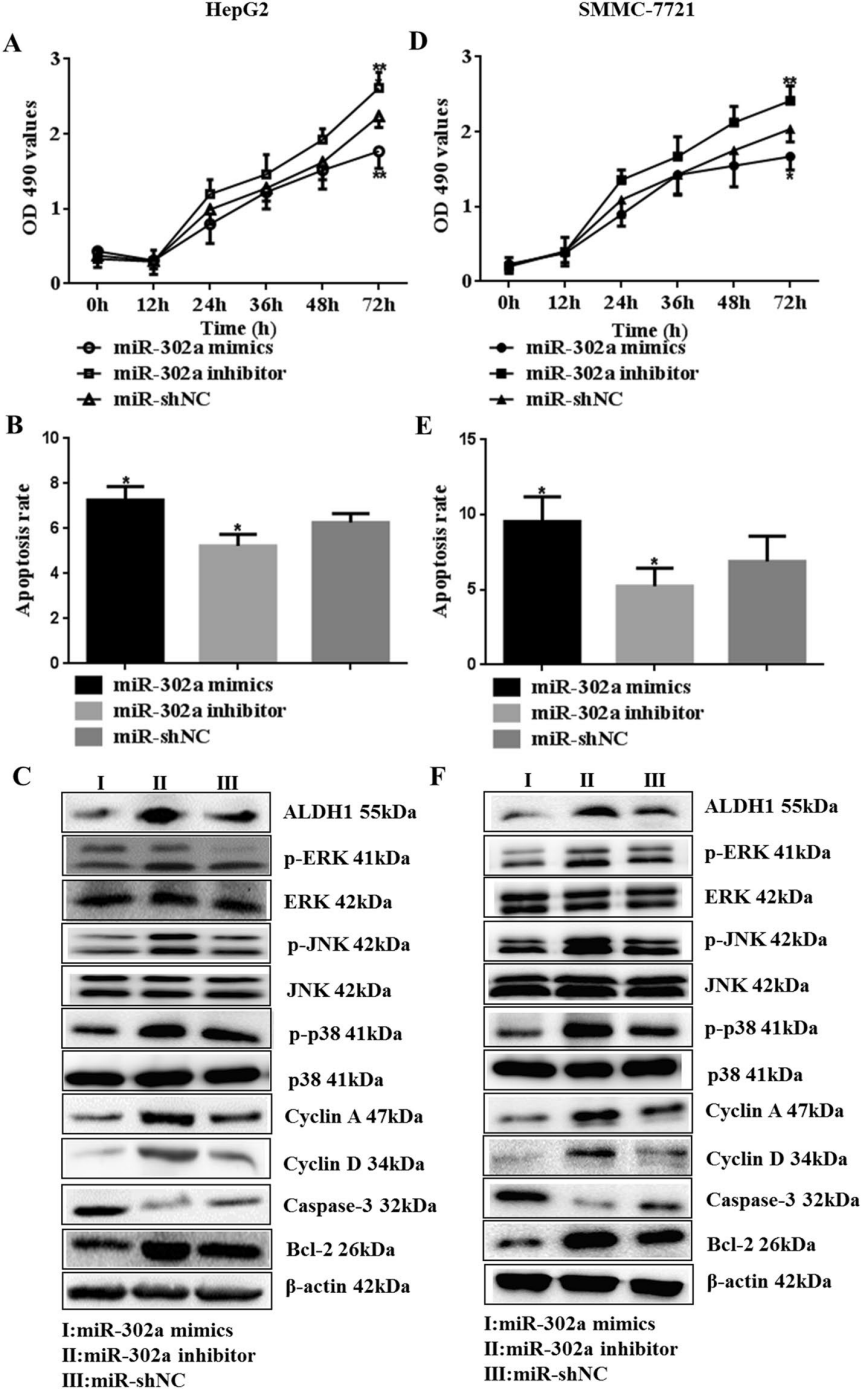
miR-302a suppresses cell proliferation and *MAPK* signaling pathway, and enhances apoptosis in hepatocellular carcinoma cells. (**A**–**C**) HepG2 cells proliferation was inhibited by miR-302a mimics. Overexpression of miR-302a increased the rate of apoptosis in HepG2 cells. Then ALDH1, p-ERK, p-JNK, p-p38, Cyclin A, Cyclin D, Bcl-2 and Caspase-3 were detected in cells that transfected miR-302a mimics, inhibitor and miR-shNC (*P < 0.05, **P < 0.01). (**D**–**F**) miR-302a vectors were transfected into SMMC-7721 cells, including mimics, inhibitor and miR-shNC. Then proliferation ability, apoptosis rate and ALDH1, Cyclin A, Cyclin D, Bcl-2, Caspase-3 and key factors in *MAPK* signaling pathway were detected in SMMC-7721 cells (*P < 0.05, **P < 0.01). Statistical analysis was conducted using Fisher’s least significant difference (LSD) method of two-way analysis of variance (ANOVA).

MTT assay results showed that miR-302a mimics could obviously inhibit HepG2 and SMMC-7721 cells viability in a time-dependent manner (Fig. 3A and D, P < 0.01) (Supplementary Tables S2 and S3). When the cells were transfected with miR-302a mimics, the apoptosis level was significantly increased (Fig. 3B and E, P < 0.01) (Supplementary Figure S3 and Table S4). In addition, the tumor marker ALDH1 and cell proliferation- and apoptosis-related factors, including Cyclin A, Cyclin D, Caspase-3 and Bcl-2, played an important role in HCC development and cell proliferation and apoptosis processes. Therefore, we examined their expression. In our western blot assay, the expression levels of Cyclin A, Cyclin D and Bcl-2 were significantly down-regulated in cells transfected with miR-302a mimics compared with cells in the miR-shNC group. Meanwhile, the Caspase-3 level was up-regulated. Moreover, miR-302a significantly reduced the expression of p-p38, p-JNK and p-ERK (Fig. 3C and F) (Supplementary Figure S4).

### Effect of *MAP3K2* and *PBX3* on *MAPK* signaling pathways

**Figure 4:**
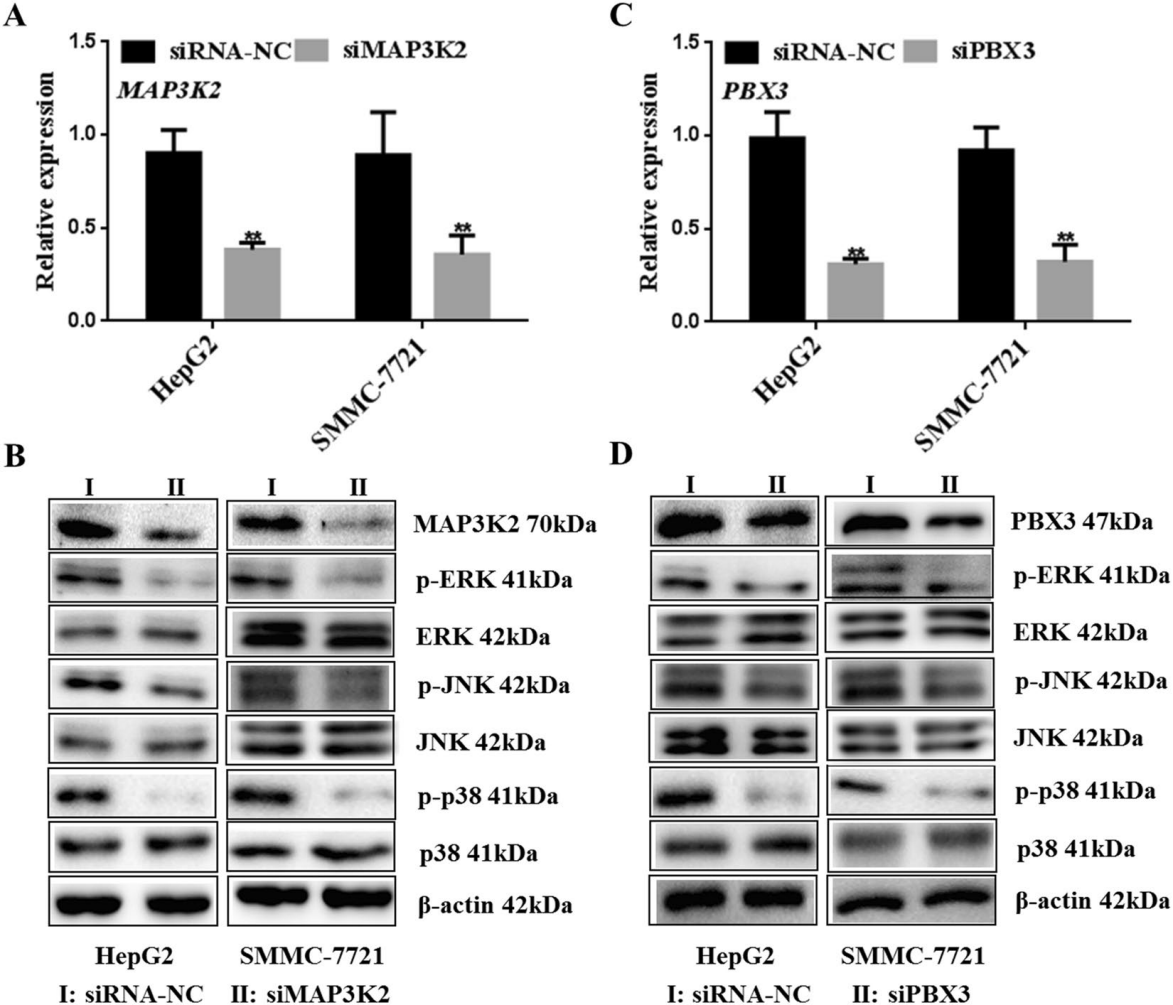
s*iMAP3K2* or *siPBX3* effects on *MAPK* signaling pathway. (**A**) *siMAP3K2* was transfected into HepG2 cells and SMMC-7721 cells. Then mRNA and protein expression were detected for interference efficiency verification (**P < 0.01). (**B**) The key factors in *MAPK* pathway were detected in HepG2 cells after *siMAP3K2* transfection, including p-ERK, p-JNK and p-p38. (**C**) The HepG2 cells and SMMC-7721 cells were cultured and transfected *siPBX3* vector (**P < 0.01). (**D**) The p-ERK, p-JNK and p-p38 were detected by western blot in cells after *PBX3* interference. Statistical analysis was conducted using Fisher’s least significant difference (LSD) method of two-way analysis of variance (ANOVA).

Since *MAP3K2* or *PBX3* are involved in regulation of the *MAPK* signaling pathway, we further verified the HCC regulation mechanism by *MAP3K2* and *PBX3* RNA interference. *MAP3K2* or *PBX3* mRNA and protein expression levels were significantly lower in *siMAP3K2-* or *siPBX3-*transfected cells (Fig. 4A and C, P < 0.01). Furthermore, *MAPK* pathway factor p-ERK, p-JNK and p-p38 also showed lower expression in cells with *siMAP3K2* and *siPBX3* treatment. The results suggested that *MAP3K2* or *PBX3* knockdown could partially inhibit the activity of *MAPK* pathway in HepG2 cells and SMMC-7721 cells. (Fig. 4B and D) (Supplementary Figure S5).

### *MAP3K2* and *PBX3* reverse the regulation effect of miR-302a on cell proliferation and apoptosis

**Figure 5:**
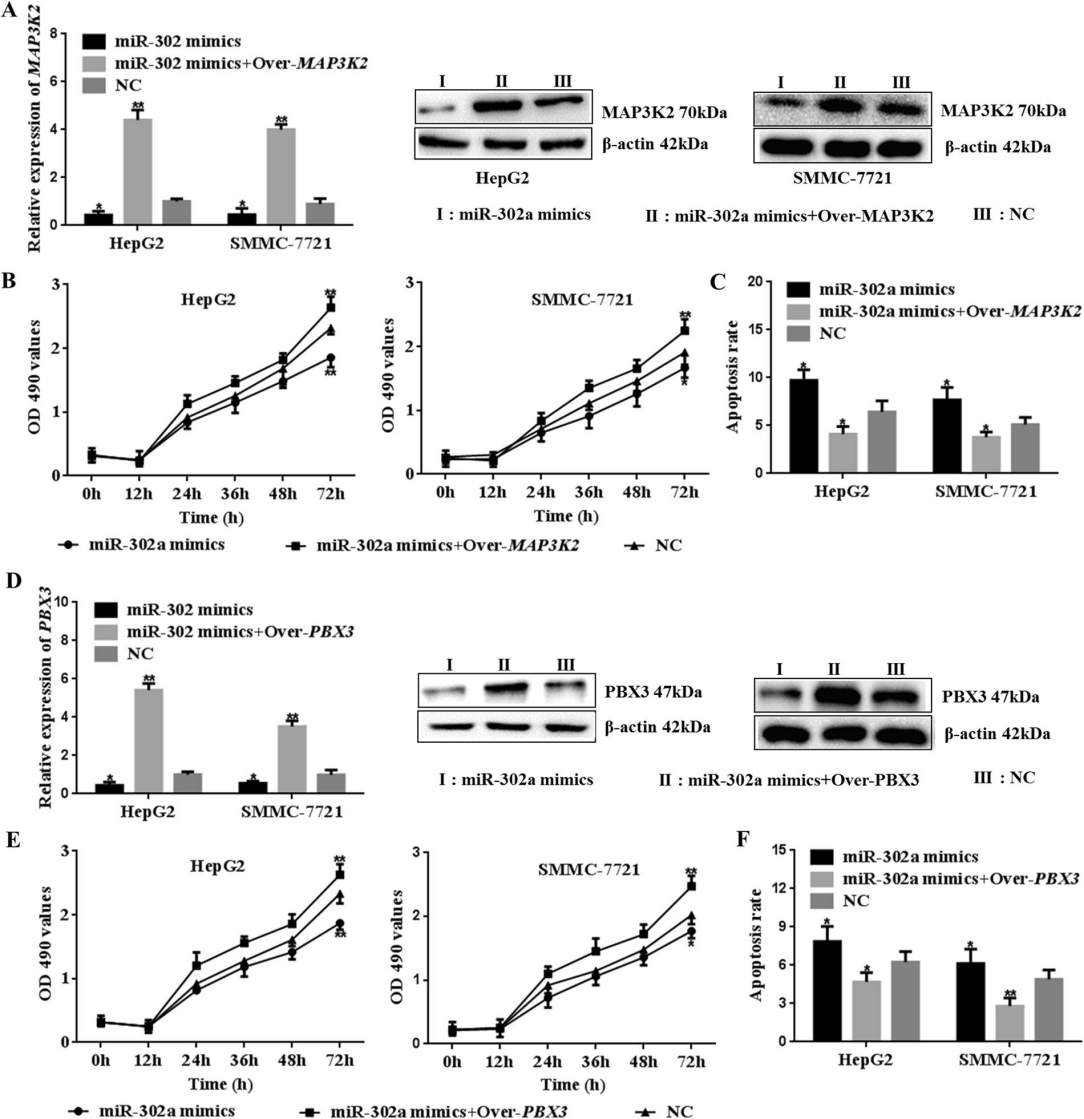
Cell proliferation ability and apoptosis rate could be rescued by *MAP3K2* or *PBX3*. HepG2 cells and SMMC-7721 cells were cultured and transfected with *miR-302a mimics*, *miR-302a mimics* + *Over-MAP3K2*, *miR-302a mimics* + *Over-PBX3* or *miR-shNC*. (**A**–**C**) Overexpression of *MAP3K2* could significantly increase the *MAP3K2* expression in cells. Then higher expression of *MAP3K2* could promote cell proliferation and reduce apoptosis (*P < 0.05, **P < 0.01). (**D**–**F**) Overexpression of *PBX3* could rescue the expression of *PBX3* in HepG2 and SMMC-7721 cells. Then higher expression of *PBX3* could promote cell proliferation and reduce apoptosis (*P < 0.05, **P < 0.01). Statistical analysis was conducted using Fisher’s least significant difference (LSD) method of two-way analysis of variance (ANOVA).

HepG2 cells and SMMC-7721 cells were transfected with *miR-302a mimics*, *miR-302a mimics* + *Over-MAP3K2* or *miR-shNC*. The results showed that *MAP3K2* mRNA and protein were higher in cells transfected with the *MAP3K2* overexpression vector (Fig. 5A, P < 0.05) (Supplementary Figure S6). Moreover, higher *MAP3K2* expression promoted cell proliferation and inhibited apoptosis in the presence of miR-302a mimics (Fig. 5B and C, P < 0.05) (Supplementary Tables S5, S6 and S9, Figure S7).

As shown in Fig. 5D, higher *PBX3* expression was observed in cells transfected with *miR-302a mimics* + *Over-PBX3* vector (P < 0.05) (Supplementary Figure S6). In addition, higher *PBX3* levels activated the cell proliferation ability (Fig. 5E, P < 0.05) (Supplementary Tables S7 and S8). Furthermore, the apoptosis rate was significantly inhibited (Fig. 5F, P < 0.05) (Supplementary Figure S8). These results indicated that *MAP3K2* and *PBX3* reversed the regulation effect of miR-302a on cell proliferation and apoptosis both in HepG2 cells and SMMC-7721 cells.

## Discussion

Previous studies have shown that miR-302a is widely involved in the proliferation, migration, and invasion of cancer cells. In osteosarcoma cells, miR-302a inhibits cell migration and invasion by directly targeting *IGF-1R*, and miR-302a can target *GAB2*, *CXCR4*, *SDF1*, *Cyclin A*, and *Cyclin D* to suppress cell proliferation, migration and invasion in glioma^12,14,31,32^. Meanwhile, miR-302a inhibits the tumorigenicity of ovarian cancer cells by suppression of *SDC1*^33^. MiR-302a exits higher expression in matched NATs. And miR-302a inhibits cell proliferation and invasion and induces cell apoptosis in hepatocellular carcinoma by directly targeting *VEGFA*^15^. In the present study, miR-302a expression was significantly lower in HCC tissues than in adjacent tissues (P < 0.01). In addition, miR-302a inhibited HepG2 and SMMC-7721 cells proliferation and promoted cell apoptosis by targeting *MAP3K2* and *PBX3*. These results were consistent with those of previous studies. Therefore, miR-302a might serve as a biomarker in HepG2 and SMMC-7721 cells. One limitation of this study is that only two of over a thousand predicted targets were assayed here because *MAP3K2* and *PBX3* could bind strongly with miR-302a. Both two genes had a close relationship with cancer deterioration. Therefore, *MAP3K2* and *PBX3* were selected from the pool of 1012 possible targets. More target genes will be validated, and the interaction between genes will be analyzed in our subsequent experiments.

In addition, GO analysis and KEGG analysis showed that *MAP3K2* can act as stress-activated protein kinase in *MAPK* signaling pathway, and also, *PBX3* can participate in the regulation of *MAPK* pathway^30^. It has been shown that MEKK2 mediates the activation of MAPKs, JNK1 and ERK^34^. Meanwhile, studies demonstrate that Src kinase activity is required for ERK activation in response to EGF, MEKK2 expression is required for ERK activation by Src, Lad and MEKK2 association is required for Src activation of ERK, and EGF and Src stimulation of ERK-regulated MEF2-dependent promoter activity requires a functional Lad-MEKK2 signaling complex^35^. In our current research, phosphorylation level of p38, ERK and JNK was down-regulated in miR-302a, *siMAP3K2* or *siPBX3* transfected cells. The results were consistent with previous research.

Multiple lines of evidence have linked aberrant *MAPK* signaling to HCC. *MAPK* signaling pathway activation is frequently reported in liver carcinoma. Studies have shown that inhibitory effect of Evo on HCC cells may be through suppressing the *MAPK* signal pathway^36^. And the findings suggest that RvD1 inhibits cell proliferation and the expression of inflammatory cytokines in LPS-treated liver cancer cells by targeting the *MAPK* pathway in HepG2 cells^25^. TGFβ1 treatment increased the phosphorylation of Smad2/3, p38 MAPK, JNK, ERK1/2, and Akt in SMMC-7721 cells and pretreatment with JR blocked TGFβ1-induced activation of Smad2/3 and Akt and MAPKs in HCC^37^. But in other research, Puerarin inhibited proliferation of SMMC-7721 cells and promoted their apoptosis in a dose- and time-dependent fashion (p < 0.05). Both the expression and phosphorylation levels of MAPK proteins were dramatically increased on puerarin treatment^38^. However, in our study, *MAP3K2* and *PBX3* could promote HepG2 and SMMC-7721 cells proliferation and inhibit cells apoptosis rate partially through achieving phosphorylation levels of MAPK proteins.

In conclusion, miR-302a regulates HepG2 cells and SMMC-7721 cells apoptosis and proliferation by targeting the *MAP3K2* and *PBX3* genes and partially through *MAPK* signaling pathways. These findings might provide novel insight into the molecular mechanisms of HCC progression and show that miR-302a is a potential biomarker.

## Materials and Methods

### Tissue samples, cell lines and vectors

HCC tissues and adjacent tissues were collected from 10 patients treated at the First Affiliated Hospital of Jilin University. Patient age ranged from 30 to 65 years old, and the cohort was evenly composed of men and women. All specimens were diagnosed by pathology (Supplementary Table S1). Then, PBS was used for cleaning, and all the samples were cut into two pieces: one was frozen for qPCR detection, and the other was immediately fixed in 4% formalin for HE staining and analysis. The study was approved by the Ethics Committee of the First Affiliated Hospital of Jilin University. All research was performed in accordance with relevant guidelines. All subjects provided written informed consent to participate in the study. L02, Bel-7402, SMMC-7721, PLC and HepG2 cells were purchased from ATCC (HB-8065, USA). Cells were cultured according to the method reference manual. Vectors for *miR-302a mimics*, *miR-302a inhibitor* and *miR-shNC* were purchased from Gene-Pharma Company in China. The *pmiR-RB-REPORT-MAP3K2/PBX3-mut*, *pmiR-RB-REPORT-MAP3K2/PBX3-WT*, *PBI-CMV3-MAP3K2*, *PBI-CMV3-PBX3*, *siMAP3K2* (*TGGATCGTATTCATATGAAG*) and *siPBX3* (*AGGTTCTTCAGATAACTCTATTG*) recombinant vectors were synthesized by GENEWIZ in China. Primers are synthesized by Sangon Biotech Company (Shanghai, China) as follows. miR-302a RT-Primer: *GTCGTATCCAGTGCAGGGTCCGAGGTGCACTGGATACGACTCACCAAA*; miR-302a upstream primer: *TGCGGTAAGTGCTTCCATGTTT*; miR-302a downstream primer: *CAGTGCAGGGTCCGAGGT*; U6 RT-Primer: *AACGCTTCACGAATTTGCGT*; U6 upstream primer: *CTCGCTTCGGCAGCACA*; U6 downstream primer: *AACGCTTCACGAATTTGCGT*; MAP3K2 upstream primer: *CCCCAGGTTACATTCCAGATGA*; MAP3K2 downstream primer: *GCATTCGTGATTTTGGATAGCTC*; PBX3 upstream primer: *ATTACAGAGCCAAATTGACCCAG*; PBX3 downstream primer: *TCTCGGAGAAGGTTCATCACAT*; GAPDH upstream primer: *ACAACTTTGGTATCGTGGAAGG*; GAPDH downstream primer: *GCCATCACGCCACAGTTTC*.

### Bioinformatics prediction

According to the paired bases and binding energy values, the bioinformatics software microRNA.org (http://34.236.212.39/microrna/home.do), miRDB (http://www.mirdb.org/), miRGen (https://omictools.com/mirgen-tool), and TargetScan (http://www.targetscan.org/vert_71/) were used to predict the target genes of miR-302a, including the *MAP3K2* and *PBX3* genes. In addition, GO analysis and KEGG analysis showed that *MAP3K2* and *PBX3* were involved in cancer regulation. Therefore, *MAP3K2* and *PBX3* were selected from the pool of 1012 possible targets.

### HE staining

The HCC and adjacent tissues were immersed in 4% paraformaldehyde (Beijing chemical plant, B0601002, China) for 3 days and transferred to 70% ethanol (Beijing chemical plant, B0301002, China). Then, the tissues were separately placed in processing cassettes, dehydrated through a serial alcohol gradient, and embedded in paraffin wax blocks. After that, 5-µm-thick tissue sections were dewaxed in xylene, rehydrated through decreasing concentrations of ethanol, washed in PBS and then stained with hematoxylin and eosin (HE). After staining, the sections were dehydrated through increasing concentrations of ethanol and xylene.

### Cell culture and transfection

Cells were detected under a microscope and then placed in an incubator. Twenty-four hours before transfection, the cells were plated at a concentration of approximately 1 × 10^6^/well into six-well culture plates with DMEM/HIGH GLUCOSE (GIBCO, 41965120, USA) containing 10% fetal bovine serum (FBS; PAA, A15-151, Austria) and 1% penicillin-streptomycin in a 37 °C humidified atmosphere with 5% CO_2_. The working concentration of penicillin and streptomycin was 100 U/mL and 100 μg/mL, respectively. The DMEM/HIGH GLUCOSE was replaced with serum-free Opti-MEM (GIBCO, Grand Island, NY, USA) when the cell confluency reached more than 80%. For luciferase activity detection, 150 µL of serum-free Opti-MEM (GIBCO, Grand Island, NY, USA) was mixed with 5 µL Lipofectamine 2000 (Invitrogen, 11668-027, USA) and 1.25 µL (20 µmol) of *miR-302a mimics* and 500 ng of *pmiR-RB-REPORT* vectors. To validate the miR-302a effects on target genes, 150 µL serum-free Opti-MEM was mixed with 5 µL Lipofectamine 2000 and 1.25 µL (20 µmol) of the *miR-302a mimics*, *inhibitor* or *miR-shNC*. To investigate the association between target genes and *MAPK* signaling pathway and their effects on human HepG2 cells and SMMC-7721, the cells were co-transfected with *PBI-CMV3-MAP3K2* or *PBI-CMV3-PBX3* and miR-302a mimics plasmids using Lipofectamine 2000 according to the manufacturer’s instructions.

### Luciferase assay

The miR-302a mimics, *MAP3K2-3′UTR-WT/PBX3-3′UTR-WT* recombinant plasmids, which contained potential target sites (*AGCACTTT*) of miR-302a, and *MAP3K2-3′UTR-mut/PBX3-3′UTR-mut* containing mutant *MAP3K2/PBX3* 3′UTR binding sites (*ACACTCCA*) were purchased from Gene-Pharma Company in China. For luciferase activity detection, the DMEM/HIGH GLUCOSE was replaced with serum-free Opti-MEM (GIBCO, 31985062, USA) when the cell confluency reached more than 80%, and 150 µL serum-free Opti-MEM was mixed with 5 µL Lipofectamine 2000 and 1.25 µL (20 µmol) of the *miR-302a mimics* and 500 ng of *pmiR-RB-REPORT* vectors for transfection. At 48 h after the transfection, the cells were harvested, and the luciferase activity was measured using a Dual Luciferase Reporter Gene Assay kit (Promega, E1960, USA) according to the manufacturer’s instructions. Three biological replicates were conducted.

### QPCR analysis

Total RNA from cells or tissues was extracted using TRIzol reagent (Invitrogen, 15596026, USA). Primer mix, miR-302a and U6 primers were designed for cDNA synthesis using a qPCR RT Kit (TOYOBO, FSQ-101, Japan). The PCR reaction solution (20 μL) was prepared according to the instructions for SYBR Premix Ex Taq (TaKaRa, DRR081A, Japan) under the following conditions: 95 °C for 1 min; followed by 40 cycles of 95 °C for 5 s and 60 °C for 30 s. *U6* and *β-actin* were used as internal controls. Three biological replicates were conducted. The data were analyzed using SPSS 20.0 software, and the 2^−ΔΔCT^ method was used according to the following formula:

$${\rm{\Delta }}{\rm{\Delta }}\mathrm{Ct}=\{{\rm{Ct}}\,({\rm{positive}})-{\rm{Ct}}\,({\rm{reference}})\}-\{{\rm{Ct}}\,({\rm{control}})-{\rm{Ct}}({\rm{reference}})\}.$$

Here, 2−ΔΔCt refers to the relative expression ratio and relative expression levels were calculated using the 2 −ΔΔCt method.

### Western blot analysis

The total protein in cells or tissues was extracted using RIPA buffer (BOSTER, AR0105, China). The protein concentration was determined using the BCA method (KeyGEN BioTECH, KGP902, China), and 40 μg of protein from each sample was separated on 10% SDS-PAGE gels and then transferred to 0.45-µm PVDF membranes (Millipore, IPVH00010, USA) with 150 mA current for 1 h. Skim milk (5%) was used as the blocking solution and incubated with the membranes at 37 °C for 1 h. Then, the membranes were incubated with *anti-MAP3K2* (1:500, ab33918, Abcam, USA), *anti-PBX3* (1:1000, ab56239, Abcam, USA), *anti-p-ERK1/2* (1:1000, ab214362, Abcam, USA), *anti-p-p38* (1:1000, ab4822, Abcam, USA), *anti-p-JNK* (1:1000, ab4821, Abcam, USA), *anti-ERK* (1:1000, ab17942, Abcam, USA), *anti-p38* (1:1000, ab31828, Abcam, USA), *anti-JNK* (1:1000, ab124956, Abcam, USA), *anti-Cyclin A* (1:1000, ab181591, Abcam, USA), *anti-Cyclin D* (1:1000, ab134175, Abcam, USA), *anti-Bcl-2* (1:1000, ab692, Abcam, USA) or *anti-Caspase-3* (1:1000, ab13585, Abcam, USA) antibody at 4 °C overnight. After 3 washes in TBST, the PVDF membrane was incubated with *goat anti-rabbit IgG* (1:3000, ab6721, Abcam, USA) for 1 h at room temperature. Finally, the protein bands were visualized using ECL Western Blotting Substrate (Invitrogen, 32109, USA).

### Cell proliferation analysis

Cell proliferation was analyzed using an MTT assay (Sigma, M2128, USA) according to the manufacturer’s instructions. HepG2 cell and SMMC-7721 cells (100 µL containing 5 × 10^3^ cells) suspensions were added to each well in a 96-well plate and incubated for 12 h. HepG2 cells were respectively transfected with miR-302a mimics, inhibitor and miR-shNC vectors using Lipofectamine 2000. For the reverse experiment, HepG2 cell and SMMC-7721 cells treated with miR-302a mimics were transfected with a *MAP3K2*/*PBX3* overexpression vector. At different time points (0 h, 12 h, 24 h, 36 h,48 h and 72 h), the culture medium was removed and replaced with culture medium containing 10 μL of sterile MTT dye (5 mg/mL). After incubation at 37 °C for 4 h, the MTT solution was removed, and 150 μL of dimethyl sulfoxide (DMSO) was added to dissolve the formazan crystals. Spectrometric absorbance at 450 nm was measured with a BioTek Synergy2 microplate photometer (BioTek, Synergy2, USA). Three biological replicates were conducted.

### Cell apoptosis analysis

Cells were harvested and reseeded in six-well plates at a density of 1 × 10^6^ cells per well after 48 h of transfection. HepG2 cell and SMMC-7721 cells were respectively transfected with miR-302a mimics, inhibitor and miR-shNC vectors using Lipofectamine 2000. For the reverse experiment, miR-302a-treated cells were transfected with MAP3K2/PBX3 overexpression vector. An Annexin V-FITC/PI apoptosis detection kit was used for apoptosis assays (Keygen Biotech, Nanjing, China). After resuspension in 400 μL of Annexin V binding buffer, the cells were incubated with 5 µL of FITC-conjugated Annexin V and 5 µL of PI for 15 min in the dark at room temperature. The early (Annexin V+/PI−) and late apoptotic (Annexin V+/PI+) cells were analyzed using a FACScan flow cytometer (BD Biosciences, San Diego, CA, USA).

### Statistical analysis

The data are reported as the mean ± standard deviation (SD). For comparison of the two study groups, statistical analysis was performed using Student’s t-test. Differences among three groups were analyzed using Fisher’s least significant difference (LSD) method or Dunnett T3 two-way analysis of variance (ANOVA) using SPSS 19.0 software (IBM Corp. Armonk, NY, USA). Values of P < 0.05 were regarded as statistically significant.

## Supplementary information

Supplementary data
