# Supplementary material for: miR-302a inhibits human HepG2 and SMMC-7721 cells proliferation and promotes apoptosis by targeting MAP3K2 and PBX3
Source: Sci Rep. 2019 Feb 14;9:2032. doi: 10.1038/s41598-018-38435-0 (PMC6375964; doi:10.1038/s41598-018-38435-0)
Supplement: Supplementary file 1 — Supplementary data [file 41598_2018_38435_MOESM1_ESM.pdf]

Title: miR-302a inhibits human HepG2 and SMMC-7721 cells proliferation and promotes apoptosis by targeting *MAP3K2* and *PBX3*

Running title: *MAP3K2* and *PBX3* are target genes of miR-302a

Authors: Meng Wang[1], Guoyue Lv[1], Chao Jiang[1], Shuli Xie[1], Guangyi Wang[1,*]

[1]Department of Hepatobiliary and Pancreatic Surgery, The First Hospital of Jilin University, 71 Xinmin Street, Changchun 130021, Jilin, China.

[*]**Correspondence to:** Guangyi Wang, MD, PhD, Department of Hepatobiliary and Pancreatic Surgery, The First Hospital of Jilin University, 71 Xinmin Street, Changchun 130021, Jilin, China. Tel: (86) 431-81875165, Fax: (86) 431-81875165; E-mail: wmgdwk3@163.com

Table S1 Clinical and pathological characteristics of the studied cohort of HCC patients

Case	Gender	Years	Grading	TNM	Background disease	HBV	HCV
1	M	69	G2	T1N0M0	active cirrhosis	+	-
2	M	58	G2	T1N0M0	active cirrhosis	+	-
3	M	35	G2	T1N0M0	active cirrhosis	-	+
4	F	63	G2	T2N0M0	active chronic hepatitis with steatosis of moderate level	+	-
5	F	66	G2	T2N0M0	cirrhosis with active chronic hepatitis	-	+
6	M	30	G1	T1N0M0	active cirrhosis	-	+
7	F	57	G1	T1N0M0	cirrhosis with active chronic hepatitis	-	+
8	F	62	G2	T2N0M0	active cirrhosis	+	-
9	M	65	G1	T1N0M0	active cirrhosis	+	-
10	F	60	G2	T1N0M0	active cirrhosis	-	+

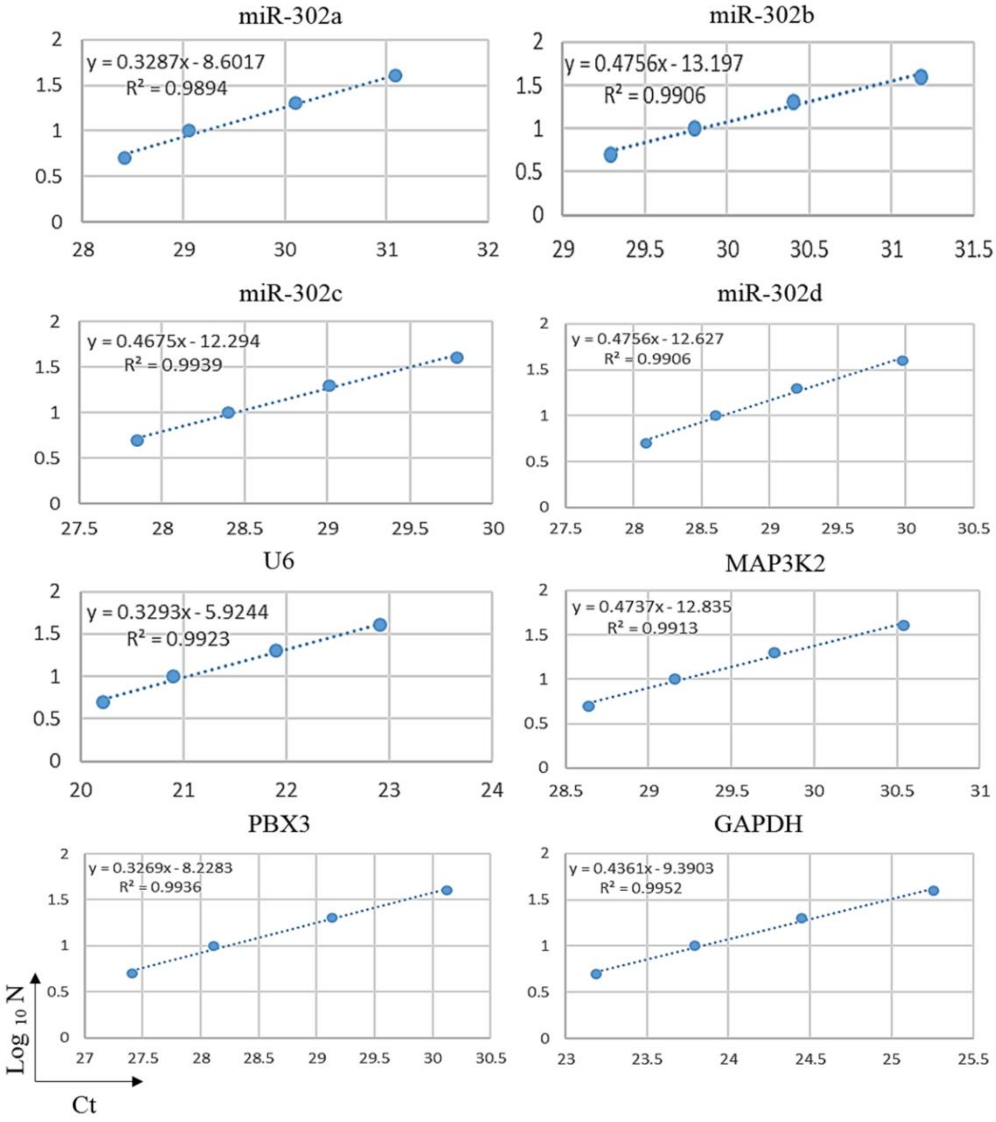

Figure S1 Validating the sensitivity and linear range of qPCR assay.

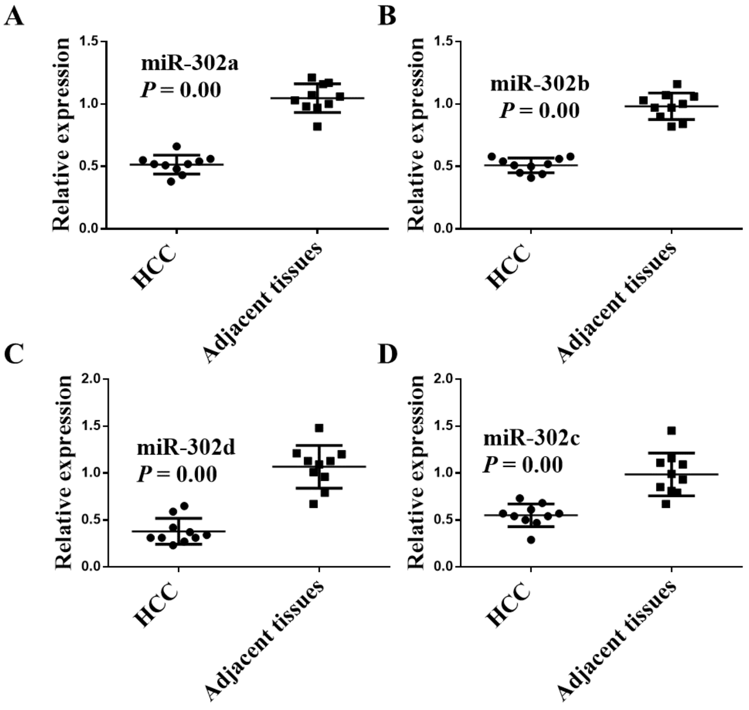

Figure S2 Relative expression of miR-302a, miR-302b, miR-302c and miR-302d in HCC and adjacent tissues.

Table S2 HepG2 cell proliferation data after miR-302a transfection

Groups	miR-302a mimics	miR-302a inhibitor	miR-shNC
Time (h)	Mean±SD	Mean±SD	Mean±SD
0	0.4333±0.1077	0.3300±0.1200	0.3767±0.1577
12	0.3100±0.1100	0.2967±0.1528	0.3033±0.1777
24	0.7967±0.2577	1.1967±0.1928	0.9900±0.2000
36	1.2233±0.2251	1.4600±0.2646	1.2767±0.1728
48	1.5167±0.2528	1.9233±0.1428	1.6200±0.2300
72	1.7700±0.2300	2.6133±0.2082	2.2367±0.1517

Table S3 SMMC-7721 cell proliferation data after miR-302a transfection

Groups	miR-302a mimics	miR-302a inhibitor	miR-shNC
Time (h)	Mean±SD	Mean±SD	Mean±SD
0	0.2243±0.1185	0.2000±0.1199	0.2177±0.1098
12	0.3800±0.1312	0.3932±0.1944	0.4031±0.1963
24	0.8956±0.1496	1.3561±0.1311	1.0891±0.2301
36	1.4211±0.2350	1.6700±0.2123	1.4267±0.2715
48	1.5439±0.2818	2.1233±0.2128	1.7500±0.1890
72	1.6900±0.1819	2.4230±0.1982	2.0352±0.1721

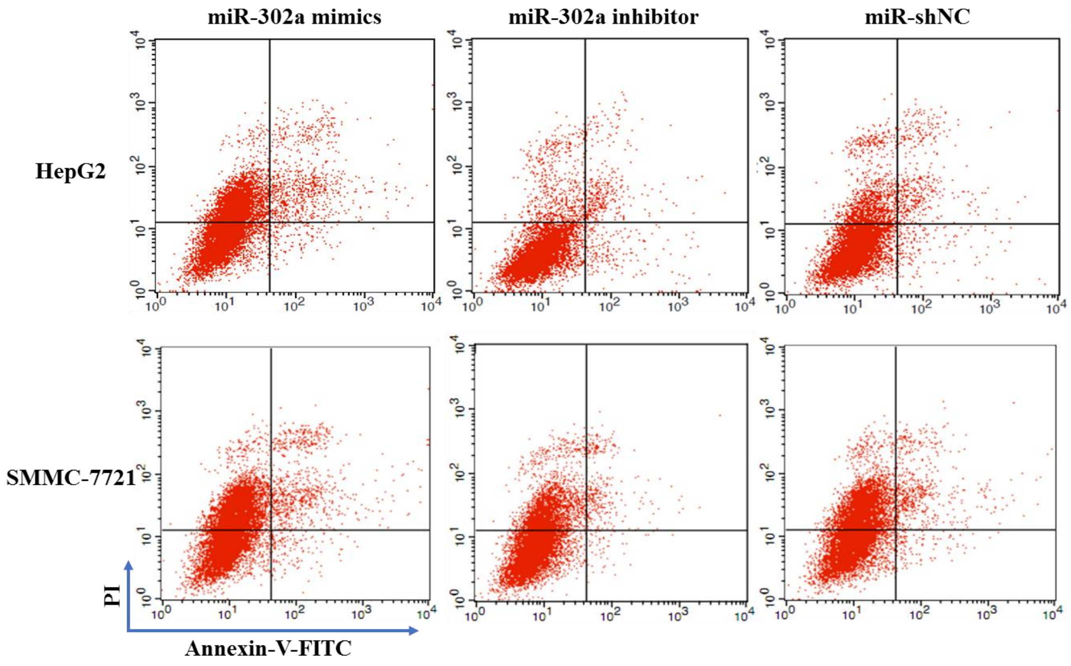

Figure S3 HepG2 and SMMC-7721 cell apoptosis rate after miR-302a transfection

Table S4 HepG2 and SMMC-7721 cell apoptosis rate after miR-302a transfection

**Groups**	**miR-302a mimics**	**miR-302a inhibitor**	**miR-shNC**
	Mean±SD	Mean±SD	Mean±SD
HepG2	7.2633±0.5976	5.2333±0.5012	6.2667±0.40415
SMMC-7721	9.5376±1.6588	5.2333±1.2012	6.8778±1.70415

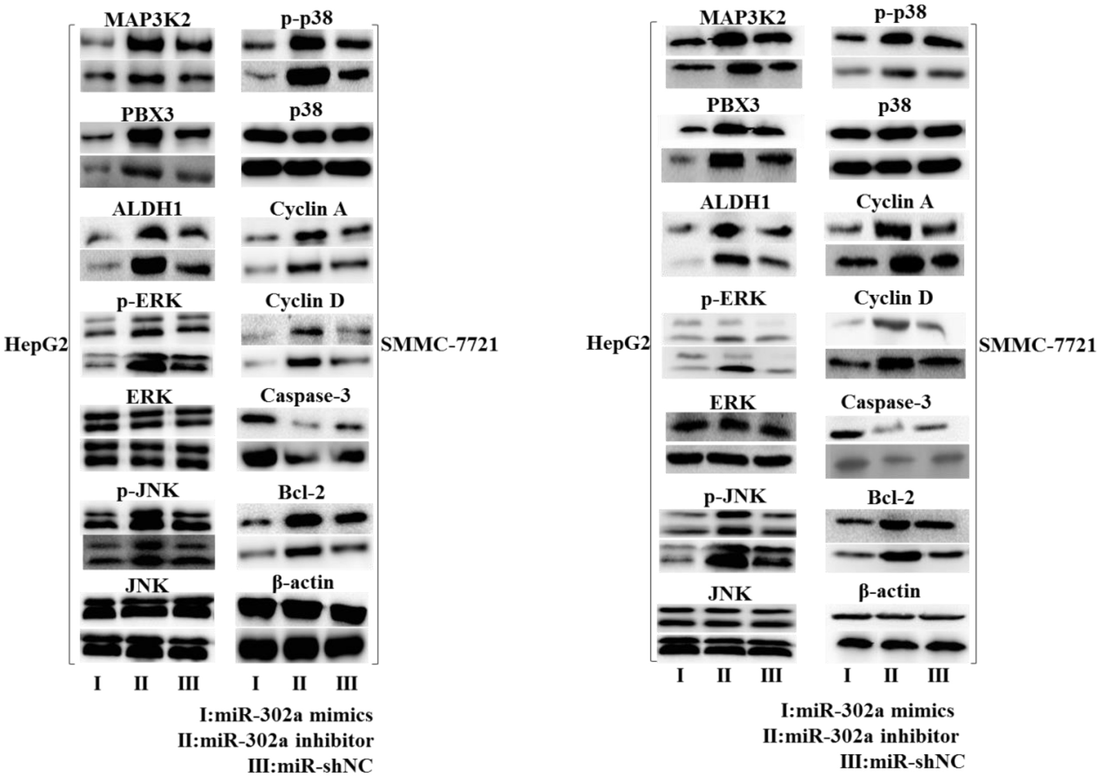

Figure S4 The effects of miR-302a on target genes and *MAPK* signaling pathway

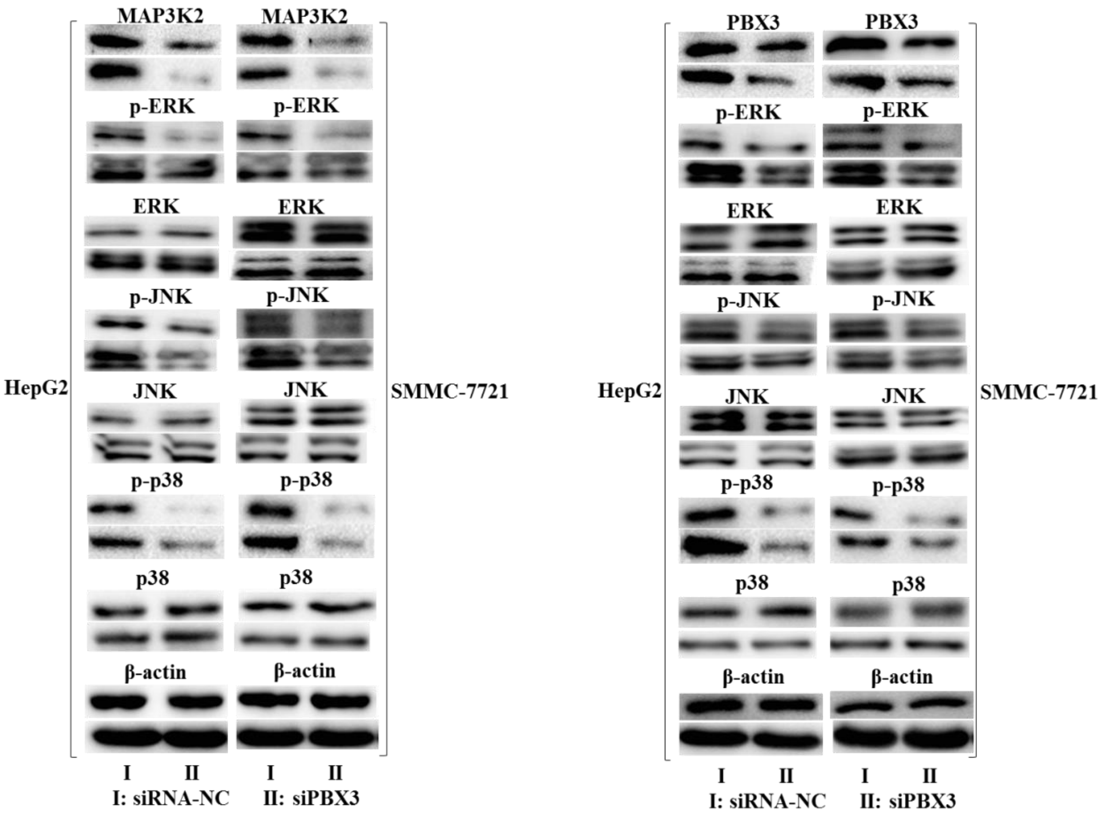

Figure S5 The effects of *MAP3K2*/*PBX3* on *MAPK* signaling pathway

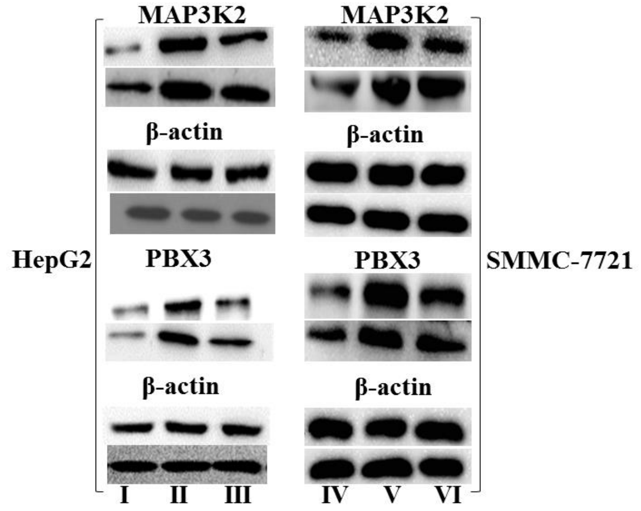

I:miR-302a mimics **IV:miR-302a mimics**

II:miR-302a mimics+Over-*MAP3K2* **V: miR-302a mimics+Over-*PBX3***

III:miR-shNC **VI:miR-shNC**

Figure S6 The protein expression of *MAP3K2*/*PBX3* after rescue experiment

Table S5 HepG2 cell proliferation data after *MAP3K2* rescue experiment

Groups	miR-302a mimics	miR-302a mimics+Over-*MAP3K2*	NC
Time (h)	Mean±SD	Mean±SD	Mean±SD
0	0.3200±0.1258	0.3300±0.1090	0.3100±0.1120
12	0.2500±0.1105	0.2367±0.1528	0.2467±0.05082
24	0.8400±0.1246	1.1300±0.1359	0.9167±0.0577
36	1.1500±0.1606	1.4567±0.1055	1.2600±0.1089
48	1.4867±0.1055	1.8200±0.1012	1.6867±0.2577
72	1.8567±0.1528	2.6367±0.1726	2.3201±0.1098

Table S6 SMMC-7721 cell proliferation data after *MAP3K2* rescue experiment

Groups	miR-302a mimics	miR-302a mimics+Over-*MAP3K2*	NC
Time (h)	Mean±SD	Mean±SD	Mean±SD
0	0.2300±0.1121	0.2551±0.1127	0.2700±0.1000
12	0.2400±0.1212	0.2122±0.1285	0.3041±0.0808
24	0.6500±0.1347	0.8344±0.1211	0.7125±0.0577
36	0.9100±0.1892	1.3521±0.1122	1.1121±0.1000
48	1.2589±0.1951	1.6570±0.1341	1.4621±0.1577
72	1.6673±0.1528	2.2451±0.1823	1.9121±0.1991

Table S7 HepG2 cell proliferation data after *PBX3* rescue experiment

Groups	miR-302a mimics	miR-302a mimics+Over-*PBX3*	NC
**Time (h)**	Mean±SD	Mean±SD	Mean±SD
0	0.3133±0.0577	0.3200±0.1095	0.3200±0.0700
12	0.2500±0.1189	0.2500±0.1055	0.2467±0.0208
24	0.8167±0.0577	1.2033±0.2082	0.9200±0.1000
36	1.1833±0.1517	1.5600±0.1101	1.2800±0.0953
48	1.4167±0.1128	1.8567±0.1517	1.6100±0.1128
72	1.8700±0.1623	2.6300±0.1646	2.3333±0.1517

Table S8 SMMC-7721 cell proliferation data after *PBX3* rescue experiment

Groups	miR-302a mimics	miR-302a mimics+Over-*PBX3*	NC
**Time (h)**	Mean±SD	Mean±SD	Mean±SD
0	0.3133±0.2133	0.2212±0.0821	0.2254±0.1221
12	0.2500±0.2341	0.2531±0.1221	0.2452±0.1311
24	0.8167±0.7235	1.1022±0.1511	0.9211±0.1082
36	1.1833±1.0611	1.4521±0.1366	1.1466±0.2016
48	1.4167±1.3576	1.7217±0.1241	1.4782±0.1521
72	1.8700±1.7701	2.4722±0.1123	2.0231±0.1646

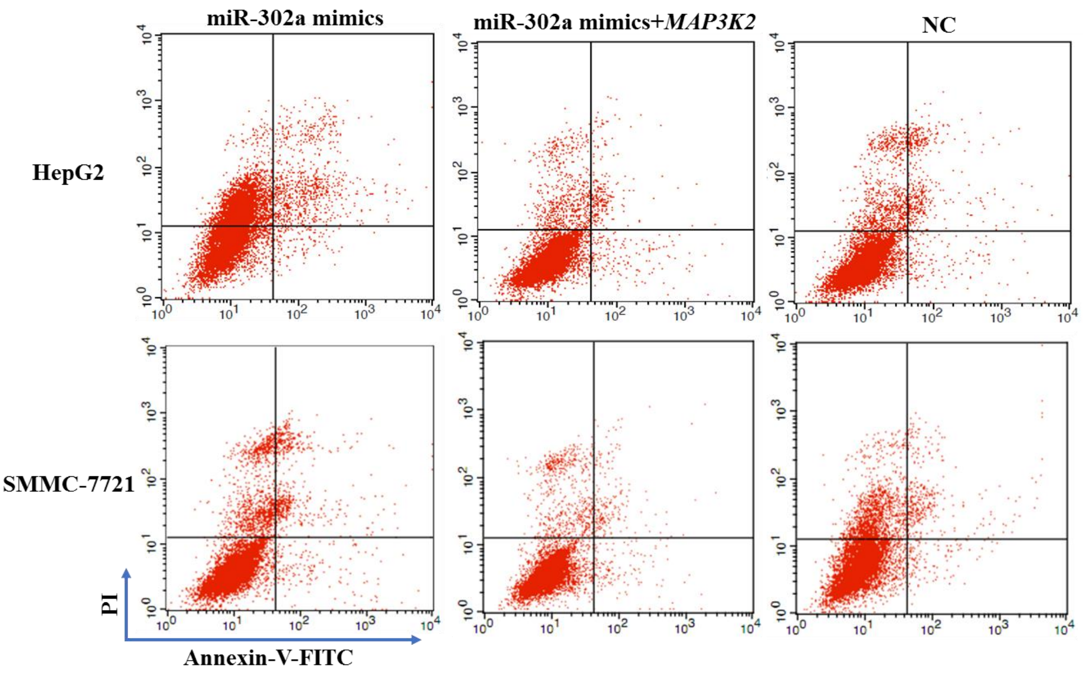

Figure S7 HepG2 and SMMC-7721 cell apoptosis rate after *MAP3K2* rescue experiment

Table S9 HepG2 cell apoptosis rate after *MAP3K2* rescue experiment

**Groups**	**miR-302a mimics**	**miR-302a mimics+Over-*MAP3K2***	**NC**
	Mean±SD	Mean±SD	Mean±SD
HepG2	9.7233±1.1069	4.1100±0.7900	6.4367±1.1457
SMMC-7721	7.6689±1.3215	3.7781±0.5672	5.1123±0.7458

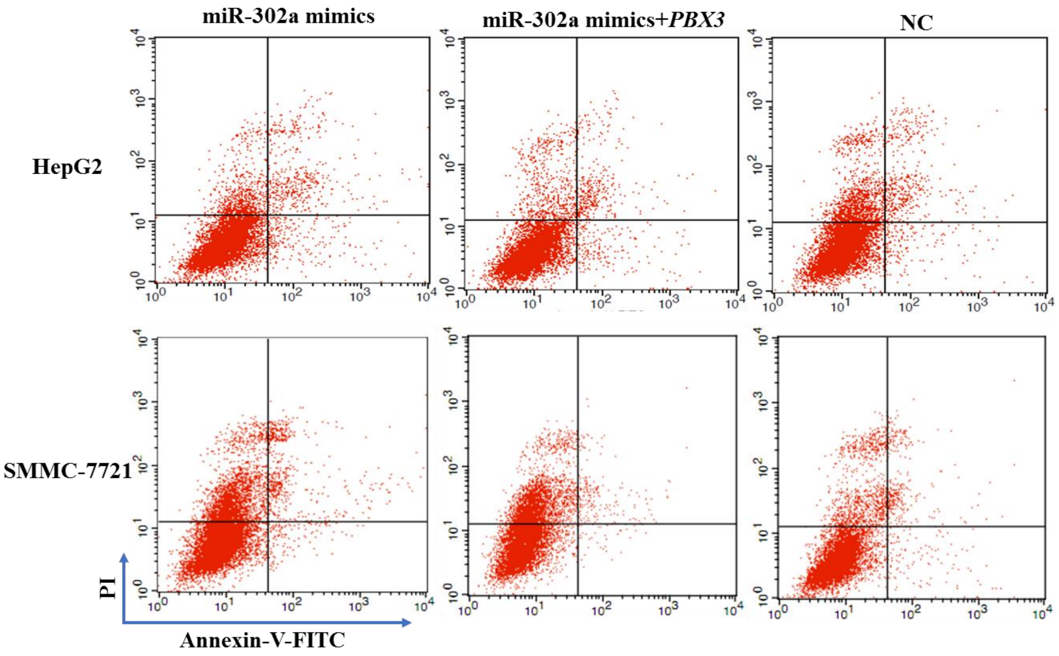

Figure S8 HepG2 and SMMC-7721 cell apoptosis rate after *PBX3* rescue experiment

Table S10 SMMC-7721 cell apoptosis rate after *PBX3* rescue experiment

**Groups**	**miR-302a mimics**	**miR-302a mimics+Over-*PBX3***	**NC**
	Mean±SD	Mean±SD	Mean±SD
HepG2	7.8633±1.1814	4.6967±0.7221	6.2467±0.8320
SMMC-7721	6.1452±1.1145	2.7893±0.6581	4.8948±0.7334
